# Optical nonreciprocity via transmissive time-modulated metasurfaces

**DOI:** 10.1515/nanoph-2022-0373

**Published:** 2022-07-14

**Authors:** Hooman Barati Sedeh, Hediyeh Mohammadi Dinani, Hossein Mosallaei

**Affiliations:** Metamaterials Lab, Electrical and Computer Engineering Department, Northeastern University, Boston, MA 02115, USA

**Keywords:** optical nonreciprocity, time-modulated metasurfaces, tunable metasurface

## Abstract

The frequency mixing property of time-modulated metasurfaces, attributed to the well-known phenomenon of temporal photonic transition, has led to several exotic functionalities in the last lustrum. Based on this concept, we demonstrate the possibility of achieving nonreciprocal responses in the near-infrared regime via combining a time-modulated platform and a static high-Q metasurface. In particular, the temporal metasurface is designed to up-convert the incident tone to the first higher-order harmonic, while the static platform is implemented to establish a filtering behavior with respect to the incident frequency. It is shown that while the receiver port acquires the transmitted signal in the forward direction, the amount of received power becomes negligible under the time-reversal scenario, which indicates the presented configuration exhibits different optical responses from opposite directions. In addition, the role of operating wavelength and the modulation frequency on the power isolation level are investigated, and it is demonstrated that by appropriate selection, the isolation level can reach −30 dB. Since this is the first time a nonreciprocal response is obtained in the near-infrared regime via a pure temporal modulation, we believe the idea of this paper can be of utmost importance in various applications, such as tunable optical isolators.

## 1 Introduction

The nonreciprocity theorem, which states the optical fields generated by a light source at a particular point in space are not the same compared to the scenario wherein the source and observation point are interchanged, is a fundamental scientific concept whose roots date back to the pioneering works of Stokes and Helmholtz in the middle of the 18th century [1–3]. Nonreciprocal devices have paved the way toward many exotic functionalities in wide branches of science, wherein one-way propagation is of utmost importance, such as full-duplex antennas [4], and signal isolation from a power supply [1], to cite a few. Although utilizing the magneto-optical effect via a static magnetic field has been the most common approach for establishing nonreciprocal responses, challenges such as bulkiness and space occupation have hindered the applicability of this method [5, 6]. Furthermore, on account of the significantly weak cyclotron frequency of free electrons and the Larmor frequency of spin precession of bound electrons in the optical regime, achieving an efficient nonreciprocal response in higher frequencies is an open challenge that needs to be addressed for the future generation of nonreciprocal devices [7–9]. In this light, the last decade has witnessed a significant growth of interest for obtaining nonreciprocal responses via exploiting nonlinear materials [10–16]. However, the need for high-intensity pump waves and poor isolation levels between the input and output ports have also limited the practicality of this approach for being utilized in realistic experimental setups. Therefore, according to the points mentioned above, it could be understood that another added layer of complexity in this field of research would be the establishment of asymmetric optical responses with a high level of isolation and via compact footprints.

Metasurfaces, consisting of a two-dimensional (2D) array of densely packed subwavelength elements, have emerged as new compact and easy-to-fabricate platforms that are competent to manipulate light in an unprecedented manner [17–19]. In sharp contrast to the conventional optical elements that rely on phase accumulation, the operation of metasurfaces relies on imparting abrupt resonant or geometric phase shifts to the scattered light, such that its wavefront could be engineered for a broad spectrum of applications, including beam deflection, focusing, and holography [17, 20]. While these ultra-thin platforms have opened up a new avenue in the optics community, their fixed functionality hinders their applicability in realistic scenarios. To overcome such a long-standing limitation of static metasurfaces and control their optical response in real-time, recently, an immense effort has been made to incorporate different thermal, mechanical, and electro-optical (EO) tuning mechanisms into these platforms [21, 22]. Among all the proposed approaches, EO-based techniques that rely on free-carrier effects in semiconductors [23, 24], and 2D materials [25] have attracted significant attention on account of their fast response time, low power consumption, and robust continuous tunability of individual unit cells. It should be remarked that while EO-based active metasurfaces are widely proposed for operating in the reflection mode, transmissive tunable metasurfaces in the near-infrared (NIR) spectrum are rarely presented due to the lack of suitable material and their low range of tunability [26]. For instance, while silicon has been the most widely utilized material for active platforms in the NIR regime, its electro-refraction coefficient is significantly reduced when it is configured into p−i−n and p−n junctions for implementation in all-dielectric metasurfaces [26–29]. As a result of such a decrement, the maximal achievable phase modulation via these materials is reduced significantly. A novel approach to address this issue is to increase the photons’ lifetime and their corresponding field confinement within the consisting resonators of the metasurface, such that a wide modulation is obtained via a slight change in the refractive index [26]. Such design criteria can be satisfied via implementing metasurfaces that are competent in supporting high-quality (high-Q) factor resonances, including the quasi-bound state in the continuum (QBIC) [30, 31], guided-mode resonances (GMR) [32–34], and anapole resonances [35–37], to name a few.

It should be remarked that while these geometrically fixed tunable platforms have enabled a myriad of applications, they cannot be utilized to establish nonreciprocal responses. This is attributed to the fact that when the incident light interacts with these quasi-static platforms, the scattered wave merely undergoes spatial photonic transition while its spectral information remains unchanged. To circumvent these limitations, recently, an increasing number of studies have focused on designing metasurfaces with time-variant properties in which time-reversal symmetry can be explicitly broken to achieve a nonreciprocal response.

These recently proposed structures, known as time-modulated metasurfaces (TMM), are another class of active platforms capable of engineering both the spectral and spatial content of the light on account of transition between different temporal and spatial photonic modes, respectively [38–41]. Such a capability will, in turn, extend the degree of light manipulation and lead to novel physical phenomena for different applications such as pulse shaping and time-reversal [42–44], dynamic beam steering [45–47], spatiotemporal light manipulation [48–50], signal amplification [51], extreme energy accumulation [52], wideband impedance matching [53], and wave camouflaging [54–56]. Moreover, it has been demonstrated that by introducing spatial variation into the temporal modulation, the time-reversal symmetry will be broken, and subsequently, nonreciprocal responses can be obtained [57–66]. This is attributed to the fact that a spatiotemporal modulation mimics a directional motion at the macroscopic scale, which breaks the symmetry of optical response by imparting different momenta to the forward and backward propagating waves under time-reversal. In sharp contrast to the conventional methods of achieving nonreciprocal responses, the nonreciprocity in TMM does not rely on heavy and bulky platforms, which makes them promising alternatives for on-chip applications. Furthermore, as opposed to nonlinear systems, the nonreciprocity in TMMs is independent of the intensity of the incident light and can be obtained for simultaneous excitation, which gives rise to several advantages in terms of power throughput and efficiency. In this perspective, the nonreciprocity of space-time photonic transitions of nonlocal guided and leaky modes have been previously exploited to achieve isolation in leaky-wave antennas, waveguides, and metasurfaces [67, 68]. However, while a spatiotemporally modulated metasurface provides isolation for different optical modes, it is impossible to achieve a nonreciprocal response via a purely temporal metasurface (i.e., spatial modulation) zero, since the scattering response becomes independent on the illumination direction. It should be mentioned that although under time-reversal, a pure TMM is capable of changing the wavelength of light along the same spatial pathway, which in turn yields the rejection of interference between transmitted and received signals by virtue of their orthogonality, the power isolation will be negligible due to the same frequency conversion performance and invariant spatial pathway under time-reversal. Therefore, a stand-alone, purely time-modulated platform can be safely classified as a reciprocal optical component. To the best of our knowledge, merely two works have been proposed to achieve nonreciprocal responses via pure temporal platforms, which are not only in the microwave regime, wherein designing a transmissive metasurface is straightforward, but also their design suffers from a complex configuration that requires other bulky optical elements such as Bragg grating [66, 69]. Therefore, in light of the points mentioned above, it is interesting to ask whether it is possible to obtain a nonreciprocal response via a purely time-modulated platform in the NIR regime while minimizing the number of the required optical elements of the configuration.

**Figure 1:**
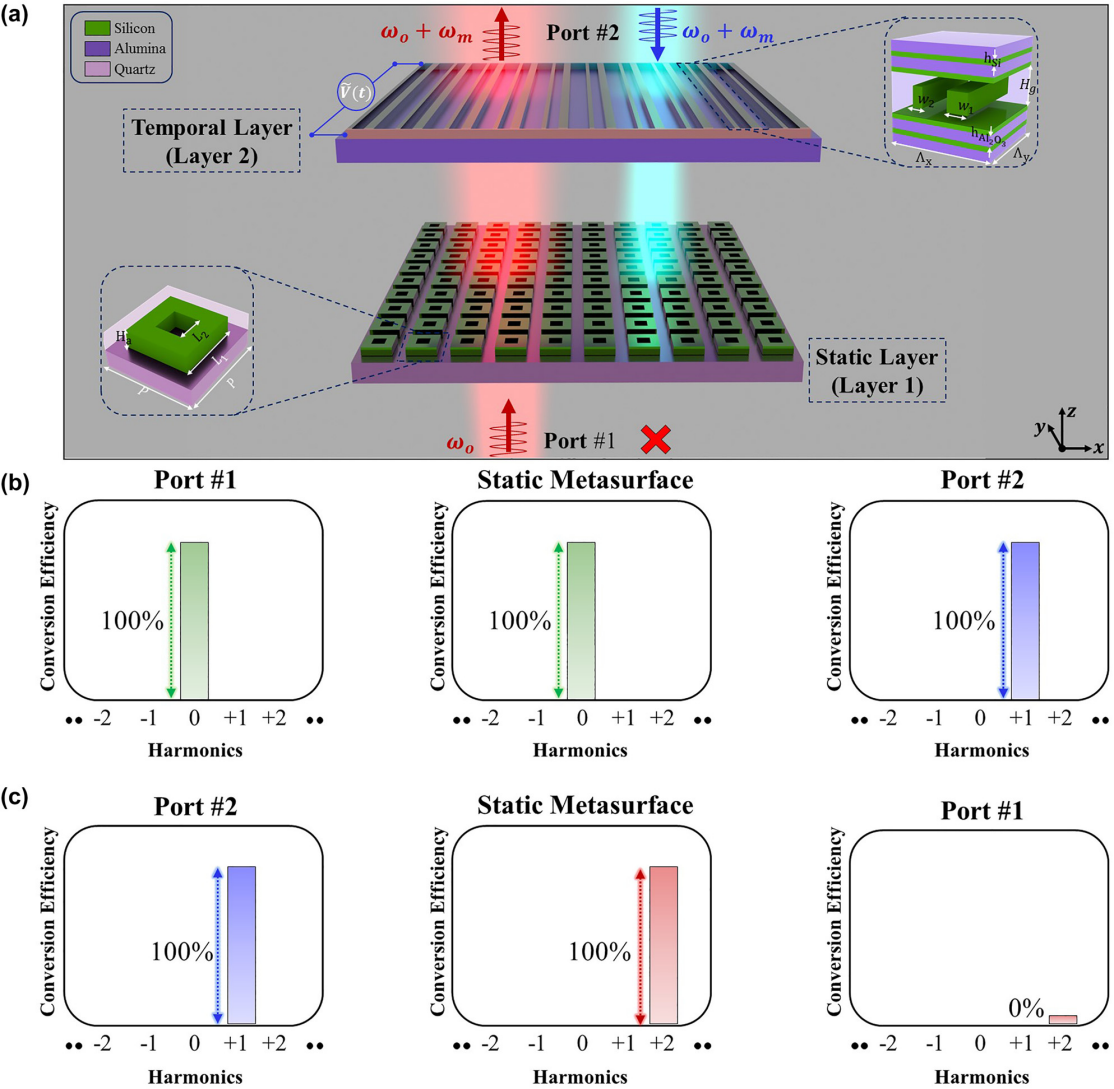
The nonreciprocity performance via transmissive metasurfaces. (a) The schematic depiction of the presented configuration that consists of two all-dielectric high-Q metasurfaces, which are under the illumination of an incident transverse electric (TE) polarized wave with its electric field pointing toward ${\hat{e}}_{x}$ axis. The constituent elements of the first layer are subjected to a temporally varying bias voltage, while the second layer is a static super high-Q metasurface. (b) The spectral evolution of the transmitted light in the forward direction at three instants of times, namely, before and after interacting with the static metasurface (light green color) and after the interaction with the pure TMM (cyan color). It is clear that while the received signal undergoes the temporal photonic transition; its amplitude remains almost the same. (c) The spectral evolution of the transmitted light under time-reversal at three instants of times, before the interaction with the pure TMM (cyan color), after passing through the temporal platform, and before interacting with the static metasurface (red color). The transmitted signal to port 1 demonstrates negligible amplitude while its spectral content shifts to higher-order frequency harmonics.

In this paper, for the first time to the best of our knowledge, we propose a purely temporal configuration in the NIR regime to achieve an asymmetric optical response that can be exploited to establish optical isolation between two channels. The provided configuration is demonstrated schematically in Figure 1a, consisting of two ultra-thin metasurfaces layers. In particular, the first layer that is competent in supporting an ultrahigh-Q factor resonance is a static metasurface consisting of 2D arrays of silicon-based nanocuboids mounted on top of a quartz substrate with square holes in their center, as shown in the inset of Figure 1a. On the other hand, the second platform, which is composed of silicon-based asymmetrical bars sandwiched between two layers of distributed Bragg reflectors (DBR), is a transmissive time-modulated metasurface capable of supporting a wide phase span (≈230°) with a high level of transmission amplitude (>60%) . While the transmissive metasurface is subjected to an optimized temporal waveform, which redirects the fundamental tone (*ω*_*o*_) into the first up-modulated frequency harmonics (*ω*_+1_ = *ω*_*o*_ + *ω*_m_, wherein *ω*_m_ represents modulation frequency), the static platform is capable of supporting a high level of transmission in a broad spectrum with an ultra-sharp dip reaching zero at *ω*_+2_ = *ω*_*o*_ + 2*ω*_m_ (the second up-modulated harmonic). It is shown that in the forward direction demonstrated with red color light in Figure 1a (i.e., transmitting from the first port to the second one with the carrier frequency of *ω*_*o*_), the received signal at the second port will undergo a temporal photonic transition (*ω*_*o*_ → *ω*_+1_), while its amplitude slightly drops. However, under time-reversal depicted with blue color light in Figure 1a (i.e., transmitting from the second port to the first one with the carrier frequency of *ω*_+1_), the transmitted wave experiences another temporal photonic transition before it reaches to the static platform (*ω*_+1_ → *ω*_+2_). On account of the particular design of the static metasurface, almost all the incident power will be reflected to the second port, which results in an isolation level more than −15 dB. While the main idea of this work is to establish isolation under normal incidence between two optical links operating at different frequencies, our idea can also be utilized in the configurations provided by Ramaccia et al. [66] and Liu et al. [69] to achieve isolation between two links with the same frequency. The presented configuration of this paper obviates the corresponding limitations associated with the previous designs based on bulky structures, which are challenging to be implemented and have efficiency limitations [70, 71].

The rest of the manuscript is organized as follows. In the next section, we will intuitively explain the core idea of our idea. Then in the third section, we will design the required elements of both mentioned metasurfaces and discuss their corresponding quasi-static results. Afterward, we will provide the temporal response of the first metasurface and evaluate the nonreciprocal efficiency of the configurations. Finally, the paper will be concluded in Section 5.

## 2 Underlying physics

On account of supporting a temporal photonic transition, the dynamic modulation of metasurfaces provides the necessary momentum for the transition of fundamental tone to higher-order harmonics defined as *f*_*n*_ = *f*_*o*_ + *nf*_m_ with *n* ∈ *Z* being an integer number denoting the order of generated frequency harmonics [45]. In sharp contrast to the static and quasi-static metasurfaces, by utilizing such a phenomenon in TMMs, known as frequency mixing property, both the spectral and spatial content of the light can be manipulated arbitrarily. The key idea to take advantage of the frequency mixing property of TMMs lies at the heart of engineering the frequency conversion performance of a time-modulated platform, which is a cascaded procedure and depends on its resonant characteristics and modulation waveform [72]. In particular, the temporal distribution of the scattered fields at the steady state is related to the desired output spectrum of frequency harmonics. In this light, the steady-state time-domain incident, transmitted, and reflected fields can be expressed as:

(1) $$\begin{array}{ll} \psi_{\text{inc}}\left(t\right) & =\psi_{o}\exp\left(i\omega_{o}t\right)\\ \psi_{\text{ref}}\left(t\right) & =\overset{+\infty }{\underset{n=-\infty }{\sum }}{\text{e}}_{n}^{\left(r\right)}\exp\left(i\left(\omega_{o}+n\omega_{m}\right)t\right)\\ & =\psi_{o}|R\left(t\right)|\exp\left(i∠R\left(t\right)\right)\exp\left(i\omega_{o}t\right)\\ \psi_{\text{tra}}\left(t\right) & =\overset{+\infty }{\underset{n=-\infty }{\sum }}{\text{e}}_{n}^{\left(t\right)}\exp\left(i\left(\omega_{o}+n\omega_{m}\right)t\right)\\ & =\psi_{o}|T\left(t\right)|\exp\left(i∠T\left(t\right)\right)\exp\left(i\omega_{o}t\right) \end{array}$$

wherein *ω*_*o*_ = 2*πf*_*o*_ and *ω*_m_ = 2*πf*_m_ are the optical and modulation angular frequencies, respectively, T(t) and R(t) are the time-domain transmission and reflection coefficients, *ψ*_*o*_ is the amplitude of incident field, and $e_{n}^{\left(t\right)}$ and $e_{n}^{\left(r\right)}$ are the complex amplitudes of transmitted and reflected fields corresponding to the *n*th frequency harmonic in the spectral domain. Moreover, to ensure the adiabatic regime of modulation, we have selected the modulation frequency to be significantly smaller than the operating one, *ω*_m_/*ω*_*o*_ ≪ 1. In particular, working in such a regime of modulation leads the phase and amplitude of the time domain reflection and transmission coefficients at each instant of time in the steady-state to be the same as those of a quasi-static metasurface working in the static regime with optical parameters corresponding to that instant of time [50]. It is noteworthy that while engineering the applied modulation waveform opens up the possibility of having control over the temporal phase of the metasurface, it will also lead to the generation of undesirable frequency harmonics on account of the variation of the amplitude with respect to the modulation signal. Such a deleterious effect can be compensated via optimizing the modulation waveform such that an optimal trade-off between the temporal phase and amplitude, whose independent control proved to be challenging, can be obtained.

In order to explain the underlying physical mechanism of the presented idea, we will first assume an ideal case wherein a transmissive time-modulated metasurface (i.e., R(t)=0 ) capable of supporting 2*π* phase span with unitary amplitude is utilized to up-convert the incident tone to the first higher-order harmonic, while the second layer is a static metasurface capable of providing a sharp optical response with its dip spectral position locating exactly at the second higher-order harmonics. In the forward case, the first port sends a signal with the frequency of *ω*_*o*_ toward the second port, as it is shown with a red color light beam in Figure 1a. Since the static metasurface is designed to transmit waves with frequencies of *ω*_*o*_ with a high level of transmission, the received signal at the second port will have the frequency of *ω*_+1_ = *ω*_*o*_ + *ω*_m_, on account of its interaction with the time-modulated platform as shown in Figure 1b. Under the time-reversal, the incident wave will be sent with the frequency of *ω*_+1_ from the second port toward the first port. Upon the interaction of light with the TMM in this scenario, the transmitted wave will be up-converted again, and its frequency will be changed to *ω*_+2_ = *ω*_+1_ + *ω*_m_ = *ω*_*o*_ + 2*ω*_m_. As it was mentioned earlier, the transmission response of the static metasurface is in such a way that it can transmit the light with the operating frequency of *ω*_*o*_, while reflecting signals with frequencies of *ω*_+2_. Therefore, under such circumstances, the transmitted light (with *ω*_+2_) will be totally reflected, while its spectral information remains unchanged, as shown with red color in Figure 1c. Eventually, the reflected signal interacts with the TMM again, and its frequency changes to *ω*_+3_ = *ω*_*o*_ + 3*ω*_m_. In this perspective, it is evident that the presented configuration exhibits different optical responses with respect to the illumination directions, which is the manifestation of a nonreciprocal response. It should be remarked that the optical response of the static layer plays a vital role in the level of isolation between two established channels.

## 3 Metasurface design

**Figure 2:**
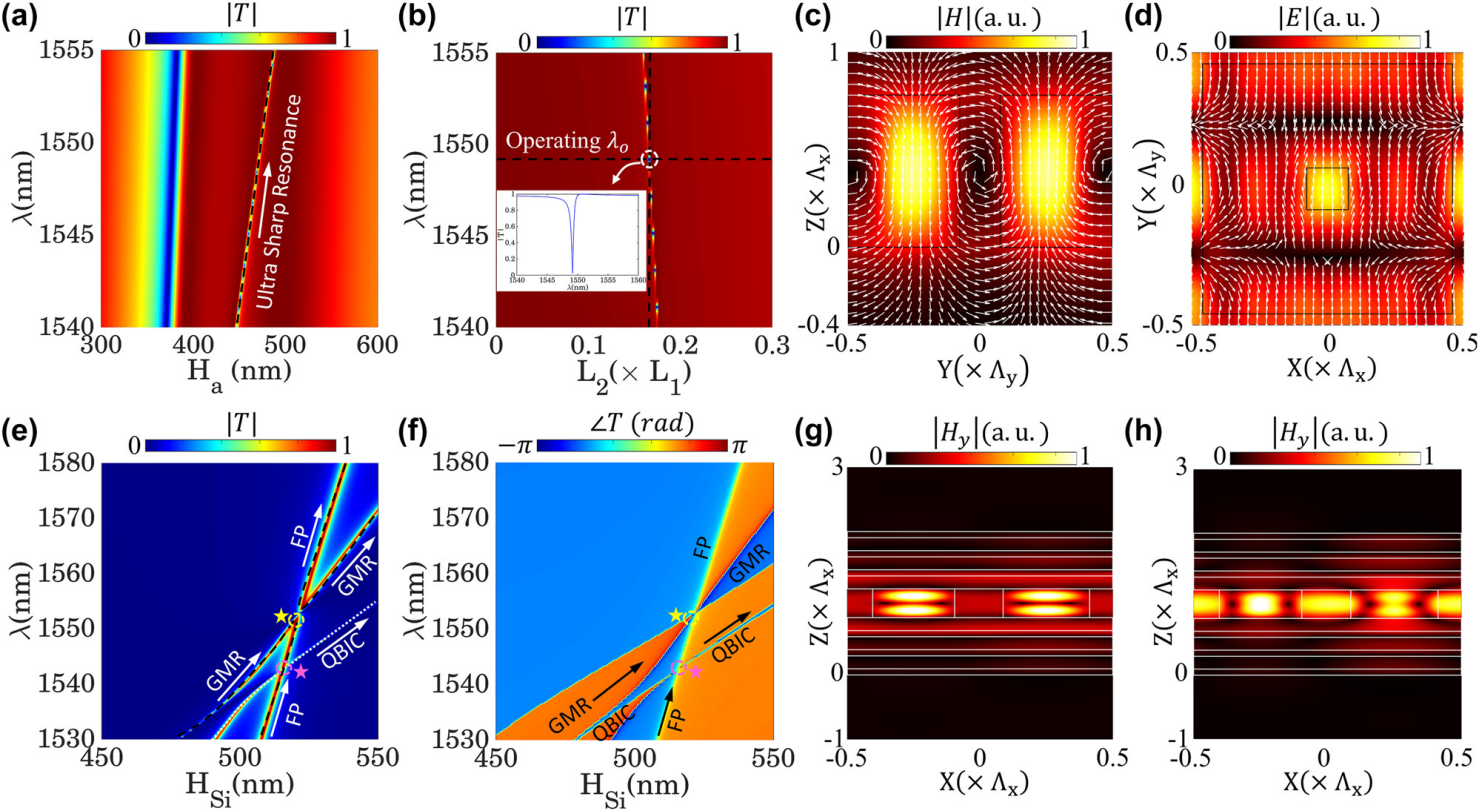
Transmission response of the static metasurface. The optical response of the ultrahigh-Q static metasurface with respect to the change in the operating wavelength ranging from *λ*_1_ = 1540 nm to *λ*_2_ = 1555 nm when (a) the thickness of the nanocuboids and (b) the length of the hollow region are varying. By fixing the structural topology of the consisting elements to be *L*_1_ = 554 nm, *L*_2_ = 0.17 × *L*_1_, *H*_a_ = 471 nm, and *P* = 603 nm, the optical response of the metasurface will demonstrate an extremely narrowband filtering in the transmission mode as it is shown in the inset of panel (b). The near-field distribution of (c) magnetic and (d) electric field within the unit cell in the (Y–Z) and (X–Y) plane, respectively. The calculated transmission (e) amplitude and (f) phase of the temporal platform as functions of its consisting elements thicknesses and operating wavelength. Increasing the height of the resonators leads to two sets of spectral overlaps between (FP-GMR) and (FP-QBIC) resonance modes. The corresponding magnetic field distributions of (g) FP and (h) GMR modes in the (*X*–*Z*) plane.

In this section, we are aiming at designing two transmissive metasurfaces that are capable of supporting the criteria mentioned above. To this end, the spectral position of the resonance dip should correspond to the second higher-order frequency harmonic (i.e., *ω*_+2_ = *ω*_*o*_ + 2*ω*_m_), such that in the time-reversal scenario (blue light in Figure 1a), the incoming wave reflected to the second port upon its interaction with the static platform. In this perspective, we designed the first layer based on a 2D array of hollow silicon nanocuboid characterized by external and internal side lengths of *L*_1_ and *L*_2_, respectively, the thickness of *H*_a_, and periodicity of *P* as it is shown in the inset of Figure 1a. It should be remarked that the 2D array is mounted on a quartz substrate, which demonstrates negligible material dispersion in the NIR spectral window and has the refractive index of *n*_quartz_ = 1.45. It is noteworthy to mention that the same material is also utilized to cover the nanocuboids such that the refractive index between substrate and superstrate matches each other. In order to investigate the effect of structural parameters (*H*_a_ and *L*_2_) on the strength, linewidth, and spectral positions of the supported resonances, we have performed two parametric studies based on rigorous coupled-wave analysis (RCWA) method, and their corresponding results are collated in Figure 2. Firstly, we have examined the effect of nanocuboids thickness on the optical response of the static layer by fixing the periodicity and lengths of the nancuboids to *P* = 603 nm, *L*_1_ = 554 nm, and *L*_2_ = 0.17 × *L*_1_, respectively, as it is shown in Figure 2a. It is evident from this figure that changing the thickness of the nano-resonators will induce two types of resonances, each with different linewidth and Q-factors. In particular, operating in the vicinity of the second resonance, whose linewidth is much narrower than its counterpart, is of utmost importance because of the reasons mentioned above. Therefore, we set the thickness of the nano-resonators to *H*_a_ = 471 nm, which ensures the existence of merely one ultrahigh-Q resonance in the transmission mode. On the other hand, as it is depicted in Figure 2b, varying the hollow region side length will directly affect the spectral location of the resonance dip, while leaving its linwidth (Q-factor) unchanged. In this perspective, in order to operate in the most common wavelength of the telecommunication band (i.e., *λ*_*o*_ ≈ 1550 nm) we have chosen the geometrical dimensions of the consisting elements to be *L*_1_ = 554 nm, *L*_2_ = 0.17 × *L*_1_, *H*_a_ = 471 nm, and *P* = 603 nm. The transmission response of the static metasurface, with the given structural parameters is provided in the inset of Figure 2b, which clearly demonstrates a high level of transmission in a broad spectrum except at a significantly narrow region locating at *λ* = 1549.2 nm. Moreover, the magnetic and electric field distributions within the consisting unit-cells of the static layer in the (*Y* − *Z*) and (*X* − *Y*) planes, are calculated and demonstrated in Figure 2c and d, respectively. It is clear from these two figures that while the magnetic field is confined around the hollow region, the electric field distributions are localized inside the nanocuboid holes. In particular, such a field confinement that controls the spectral position and strength of the resonant dip is directly attributed to the existence of the hollow region [73]. As it will be demonstrated in the following sections, such a filtering behavior plays a crucial role for establishing a nonreciprocal response via a purely time-modulated platform.

The second metasurface, which serves as a temporal platform, consists of an array of asymmetrical silicon nanobars that are sandwiched between two distributed Bragg reflectors (DBR) and have the periodicities of Λ_*x*_ = Λ, Λ_*y*_ = Λ/2 the height of *H*_*g*_, and width of *w*_1_ and *w*_2_ as it is shown in the inset of Figure 1a. The DBR Contains three quarter-wave stacks of silicon and alumina (Al_2_O_3_) with the thicknesses of *λ*_*o*_/4*n*, wherein *n* represents the refractive index of the corresponding materials, which have been obtained from experimentally measured data [74], and *λ*_*o*_ is the operating wavelength. It should be remarked that the grating height can be decomposed into the nanobar and DBR thicknesses as $H_{g}=H_{\text{Si}}+2\lambda_{o}/4n_{{\text{Al}}_{2}{\text{O}}_{3}}$ , while the width of the second nanobar, *w*_2_, can be expressed in terms of *w*_2_ = *w*_1_ − *δ*, wherein *δ* = 20 nm represents the asymmetric parameter. In this perspective, the transmission amplitude and phase of the second platform under the normal illumination of a transverse electric (TE) polarized beam with its electric field directed along the ${\hat{e}}_{x}$ direction are obtained via RCWA, and their results are depicted in Figure 2e and f, respectively as functions of the nanobar thickness (*H*_Si_) and operating wavelength. As it is evident from the provided figures, upon breaking the in-plane symmetry (i.e., *δ* ≠ 0) three resonant modes emerge, which can be identified as the quasi-bound state in the continuum (QBIC), Fabry–Pérot (FP), and guided-mode resonance (GMR). To further clarify the nature of the occurred resonances, we have also calculated the near-field distributions of the normal component of the magnetic field (|*H*_*y*_|) in Figure 2g and h at the (*X* − *Z*) plane for the FP and GMR resonances, respectively. In particular, while the field distribution of the FP mode clearly demonstrates the concentration of the optical fields at the cavity between two DBRs, the field distributions of the GMR exhibit strong enhancement within the nano-resonators.

It is clear that changing the height of the nanobars will subsequently shift the spectral positions of the emerged resonance modes on account of the change in the effective refractive index. However, the spectral position of the Fabry–Pérot mode experiences more shift due to the modification of the cavity size between top and bottom DBRs. Further increment of *H*_Si_, leads to two sets of spectral overlaps between (FP-GMR) and (FP-QBIC) resonance modes, shown with yellow and pink color stars in Figure 2e and f, which subsequently establish a Huygens-like regime at two distinctive wavelength and thicknesses. It should be remarked that such a Huygens-like behavior is attributed to the fact that each mode experiences a spectral phase shift of *π* at each excited resonance [75]. In other words, upon the spectral overlap of each pair of resonances, the spectral phase span can reach up to 2*π* while its amplitude can remain close to 100% in the transmission mode, which is a manifestation of a Huygens regime. In order to operate in the vicinity of the telecommunication band, we have set the geometrical dimensions of the second metasurface to *w*_1_ = 380 nm, *H*_g_ = 520 nm, and Λ = 2 × 580 nm, which gives rise to two sets of resonances that occur at *λ*_1_ = 1549.6 nm, and *λ*_2_ = 1544.6 nm. As it is evident from Figure 2e and f, the first resonance, originated from the overlap of FP and GMR modes, has a Huygens-like behavior (i.e., unitary transmission with 2*π* phase span), while the second resonance corresponds to the QBIC mode. Therefore, the presented metasurface can also be utilized for dual-band operation in NIR regime.

## 4 Quasi-static and temporal response

**Figure 3:**
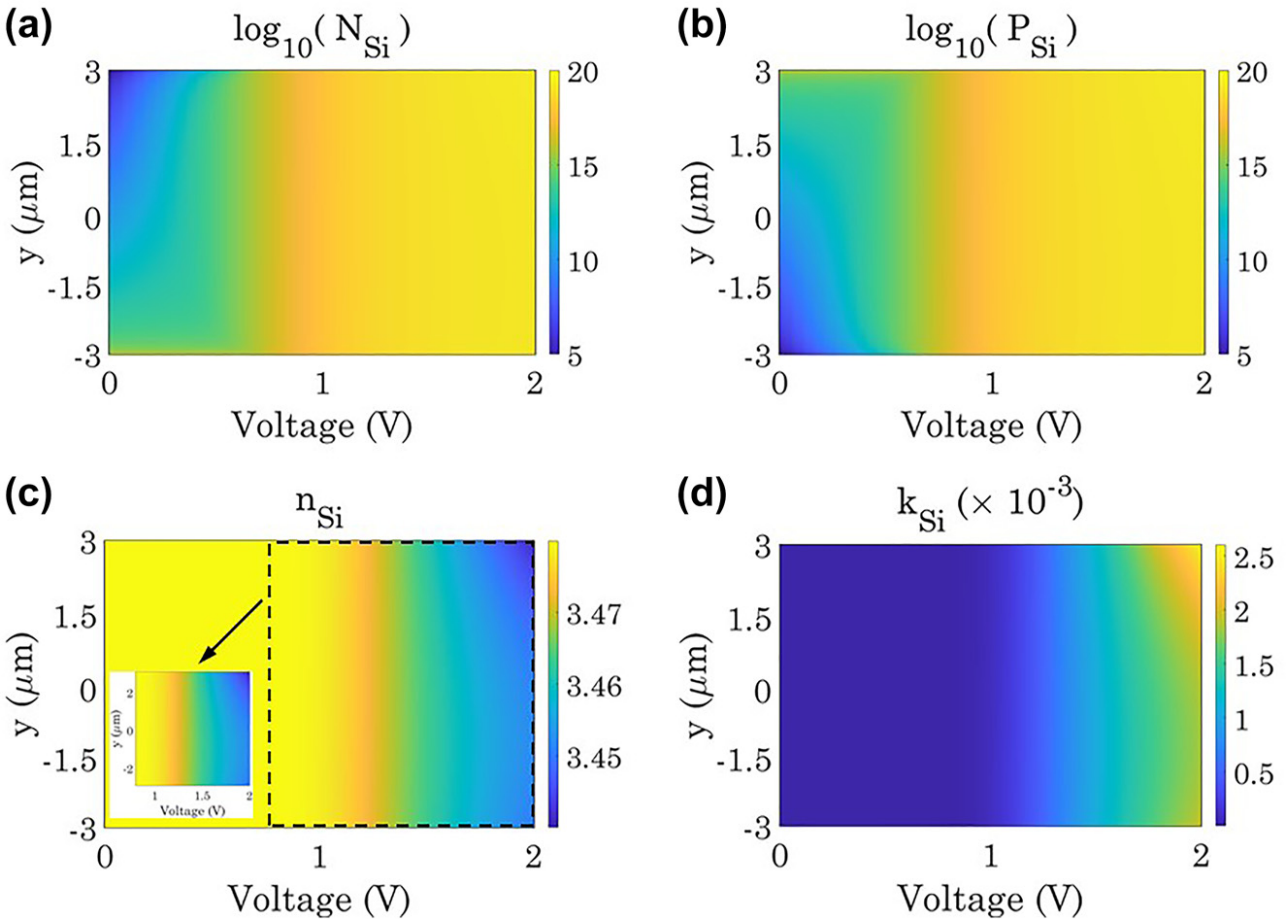
Carrier density distributions with respect to the applied voltage. The calculated (a) electron and (b) hole carrier densities as functions of position and applied bias voltage across the intrinsic region in the logarithmic scale. When the applied bias voltage is lower 0.6 V, both the electron and hole distribution inside the intrinsic region experience significant exponential changes in the vicinity of highly *N* and *P* regions. The calculated (c) real and (d) imaginary part dependency of the silicon refractive index on the spatial position and applied bias voltage along the intrinsic region. When the applied bias voltage is 0.6 V < *V* < 1.2 V, the refractive index undergoes −0.01 change in its real part, while its imaginary part varies negligibly.

**Figure 4:**
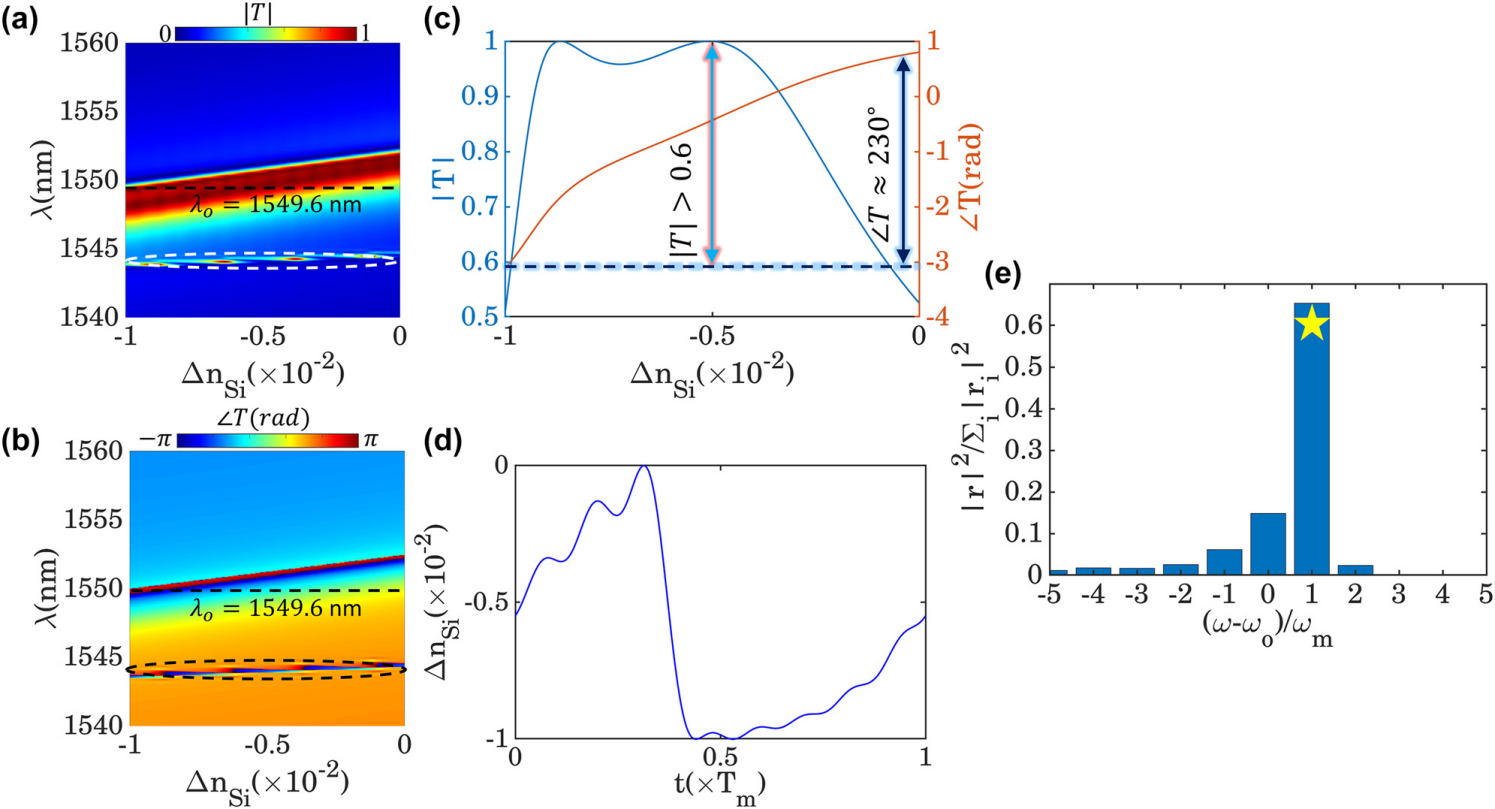
The active and time-modulated response of the second layer metasurface. The calculated transmission (a) amplitude and (b) phase of the temporal layer with respect to the operating wavelength and silicon refractive index change. Since the presented design is capable of supporting two types of resonances, its tunable response can also function in dual-bands around *λ*_1_ ≈ 1549.6 nm and *λ*_2_ ≈ 1544.6 nm as shown with black dashed lines. (c) The optical response of the same metasurface at the operating wavelength of *λ*_*o*_ = 1549.6 nm, which exhibits high transmission amplitude above 60% and phase modulation of ≈230° . (d) The applied temporal waveform to the metasurface such that it can redirect the fundamental tone to the first up modulated higher-order harmonics. (e) The calculated output spectrum of the temporal metasurface. It is evident that the designed metasurface is competent in converting the fundamental harmonic to the first up modulated harmonic with a high conversion efficiency of |*t*_+1_|^2^/*∑*_*l*_|*t*_*l*_|^2^ ≈ 70%.

As mentioned earlier, the working principle of the presented setup for establishing asymmetric optical response is based on the frequency mixing property of the TMM (i.e., the second layer) and the filtering response of the static metasurface (i.e., first layer). To this aim, the quasi-static response of the temporal platform should be obtained via changing the refractive indices of the consisting elements. In this perspective, the refractive indices of the asymmetrical nanobars are electrically modulated via p−i−n junction configuration. The same as that of our previous work [75], while the *N* and *P* doped regions are located at the two ends of the nanobars, with the length of *L*_*n*_ = 0.6 μm and *L*_*p*_ = 0.4 μm and carrier concentration of *n* = 4.5 × 10^20^ cm^−3^ and *p* = 1 × 10^20^ cm^−3^, the length and carrier concentration of the intrinsic region are fixed to *L*_*i*_ = 6 μm and 10^10^ cm^−3^, respectively. By changing the applied bias voltage from 0 to 2 V, the electron and hole carrier distributions inside the intrinsic region are calculated via Lumerical device solver, which self-consistently evaluates the Poisson and drift-diffusion equations, as demonstrated in Figure 3a and b in logarithm scale, respectively. As it is evident from these figures, while for the applied bias voltages of *V* < 0.6 V, both the electron and hole carrier densities inside the intrinsic region undergo significant exponential changes in the vicinity of highly *N* and *P* doped regions, for the greater applied voltages (*V* > 0.6 V), the carrier densities remain almost uniform across the intrinsic region. To investigate how such a carrier density distribution affects the refractive index of the asymmetrical nanobars, we employ the silicon plasma-Drude model as $n_{\text{Si}}\left(\omega \right)+ik_{\text{Si}}\left(\omega \right)=\sqrt{\epsilon_{\text{Si}}^{\left(0\right)}\left(\omega \right)-\left(e^{2}/\epsilon_{0}\omega \right)\cdot \left(\frac{P}{m_{P}\omega +ie/\mu_{P}}+\frac{N}{m_{N}\omega +ie/\mu_{N}}\right)}$ , wherein $\epsilon_{\text{Si}}^{\left(0\right)}\left(\omega \right)$ is the undoped permittivity of silicon, *μ*_*N*_ = 80 cm^2^ V^−1^ S^−1^ and *μ*_*P*_ = 60 cm^2^ V^−1^ S^−1^ are the electron and hole mobilities, *m*_*N*_ = 0.27*m*_0_ and *m*_*P*_ = 0.39 *m*_0_ are is electron and hole effective masses, while *e* and *m*_0_ represents the electron charge and rest mass, respectively [76]. In this perspective, the nanobars refractive index dependency on the applied voltage is calculated from the obtained data of Figure 3a and b, and its results are collated in Figure 3c and d. As it is clear from these panels, when the applied bias voltage is lower than 0.6 V, which corresponds to the rapid change in the electron and hole carrier densities, the real and imaginary parts of the silicon refractive index change negligibly. However, when the applied bias voltage increases beyond 0.6 V and lower than 1.2 V, which corresponds to the uniform carrier densities of log (*N*) = log (*P*) = 18.7, the real part of the refractive index varies from 3.48 to 3.47 (Δ*n*_Si_ = −0.01) while its imaginary part increases from 0 to 0.5 × 10^−3^. It should be remarked that when the applied bias voltage is greater than 1.2 V, the complex refractive index of the asymmetrical nanobars alters significantly, leading to nonuniformity in the intrinsic region. Therefore, we set the applied voltage to vary in the range of 0.6 V <V<1.2 V, such that the electron and hole carrier densities change uniformly (Δ*N* = Δ*P* = 5 × 10^18^ cm^−3^, or log (N) = log (P) = 18.7) across the intrinsic region, giving rise to the refractive-index change of Δ*n*_Si_ = −0.01. Following the points mentioned above, the corresponding transmittance amplitude and phase of the designed metasurfaces are calculated as functions of operating wavelength and silicon refractive index change as shown in Figure 4a and b. As it is evident from these figures when Δ*n*_Si_ increases from negative to positive values, the spectral position of the transmission resonance shifts toward longer wavelengths. Moreover, at the operating wavelength of *λ*_*o*_ = 1549.6 nm, a slight change in Δ*n*_Si_ from −0.01 to 0, leads to a top-flat amplitude above 60% and phase span of 230° as it is demonstrated in Figure 4c. It should be mentioned that because the presented metasurface is capable of supporting two types of resonances (i.e., Huygens like and QBIC), its tunable response can also operate in dual-band as it is shown in Figure 4a and b) with black dashed lines. To the best of our knowledge, such a dynamic dual-band design has the best trade-off between the amplitude and phase modulation, compared to works presented in the literature [21, 22].

As mentioned earlier, the main idea of this paper lies at the heart of the frequency mixing property of TMMs. To this aim, the frequency conversion performance of a time-modulated metasurface should be obtained via determining the temporal evolution of the steady-state amplitude and phase, which depend on the applied modulation waveform. In this perspective, utilizing an evolutionary algorithm for optimizing the modulation waveform leads to an optimal response since the correlation and trade-offs between phase and amplitude will be taken into account. For this purpose, we have expressed the temporal dependency of the silicon refractive indices in terms of a truncated Fourier series as $n\left(t\right)=n_{o}+\sum_{l=0}^{l=8}a_{n}\sin\left(n\omega_{m}t\right)+b_{n}\cos\left(n\omega_{m}t\right)$ wherein *n*_*o*_, *a*_*n*_, and *b*_*n*_ are the coefficients which ought to be optimized by employing genetic algorithm (GA) to arrive at the desired spectral diversity of frequency harmonics. In this paper, the goal is to convert the incident power at the fundamental tone to the first-order up-modulated frequency harmonics (i.e., *ω*_*o*_ → *ω*_*o*_ + *ω*_m_) while minimizing the conversion to other frequency mixing products. To this end, we have defined the objective of the optimization as maximizing the conversion efficiency of the first-order up-modulated frequency harmonics, which can be expressed in terms of the transmitted power residing at *ω*_*o*_ + *ω*_m_ over the total transmitted power as |*t*_+1_|^2^/*∑*_*l*_|*t*_*l*_|^2^, wherein |*t*_*l*_| denotes the transmission coefficient corresponding to the *l*th frequency harmonic. The optimized refractive index modulation waveform and its corresponding normalized output frequency spectrum are depicted in Figure 4d and e, respectively. As evident from panel (d), the optimized temporal refractive index demonstrates a sawtooth-like behavior with broken time-reversal symmetry, which is in good agreement with the phase modulation prescription for serrodyne frequency translation that requires a sawtooth temporal profile [45, 72]. Furthermore, the calculated normalized output spectrum clearly shows that a significant portion of the transmitted power is dwelling at the first up-modulated frequency harmonics (yellow star), yielding a normalized frequency conversion efficiency of |*t*_+1_|^2^/*∑*_*l*_|*t*_*l*_|^2^ ≈ 70%. It is noteworthy to mention that the non-unity conversion efficiency and the existence of other higher-order harmonics is attributed to the limited quasi-static phase span and nonuniform quasi-static amplitude across the phase modulation range. In particular, a unity conversion efficiency can be obtained merely for a tunable metasurface capable of providing 2*π* phase with a uniform amplitude at the quasi-static operation [45]. Although fulfilling such criteria is of utmost difficulty in the higher frequency regimes such as NIR, the expeditious growth in material science and rapid emergence of new 2-D materials with improved characteristics have shown great potential for achieving better performance in the near future.

**Figure 5:**
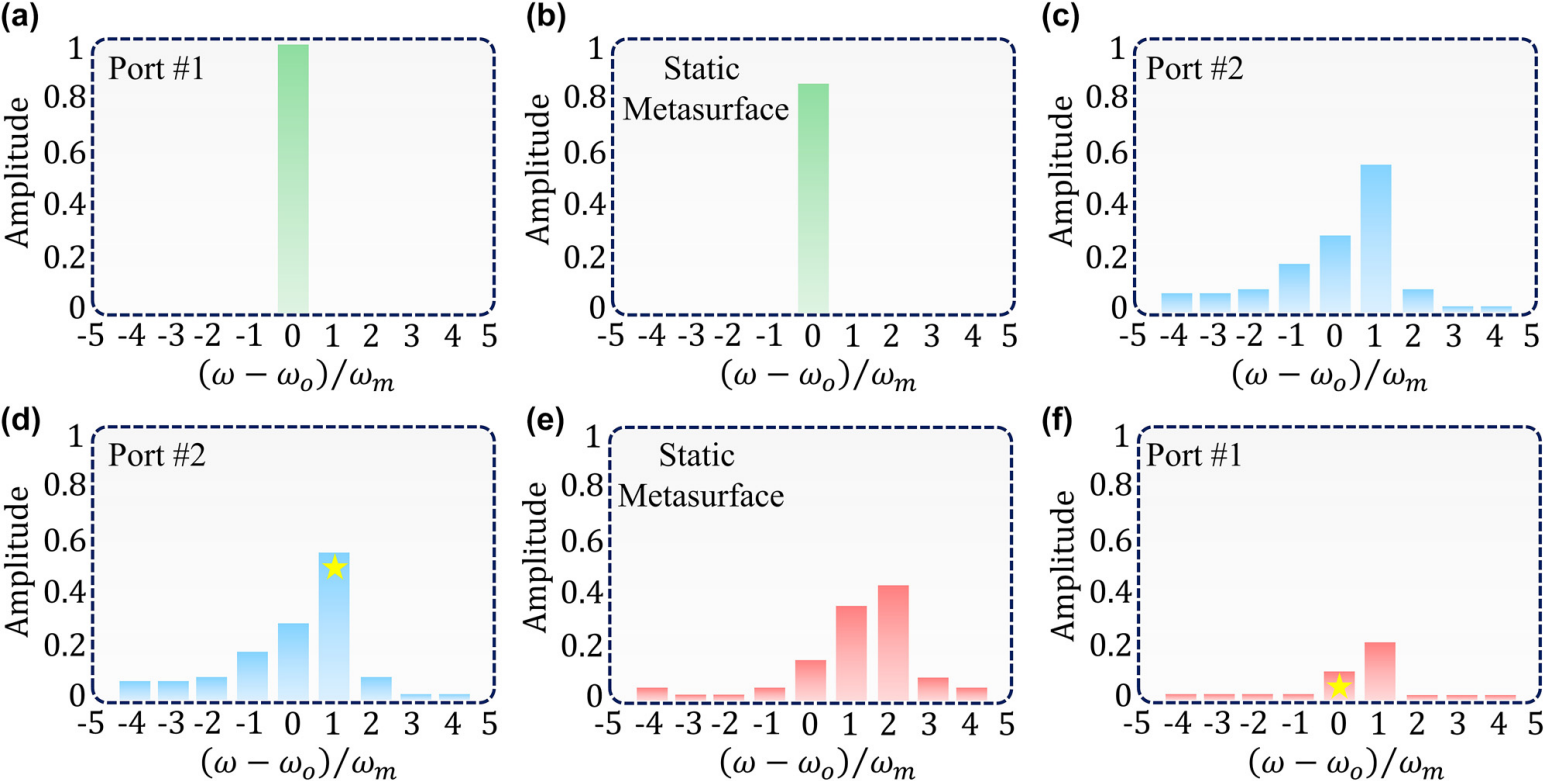
The nonreciprocal performance of the presented configuration. The spectral evolution of the (a) incident (b) transmitted signal prior to and after its interaction with the static layer. It is clear that while the signal amplitude slightly decreases, its spectral information remains untouched (c, d) the calculated output frequency spectrum of the signal after interacting with the time-modulated platform. In this case, both the amplitude and spectral content of the light will be manipulated. (e) The derived spectral information of the light after passing through the time-modulated metasurface in the time-reversal scenario. Owing to the second temporal photonic transition, the spectral distribution of the light will be changed once again. (f) The output spectrum of the signal that reaches the first port. On account of the special design of the static layer and its filtering behavior, the components of the light whose frequencies fall within the range of *ω*_*o*_ ± *ω*_m_ and *ω*_*o*_ ± 2*ω*_m_, will be totally reflected, leaving a negligible amount of signal to reach to the first port.

**Figure 6:**
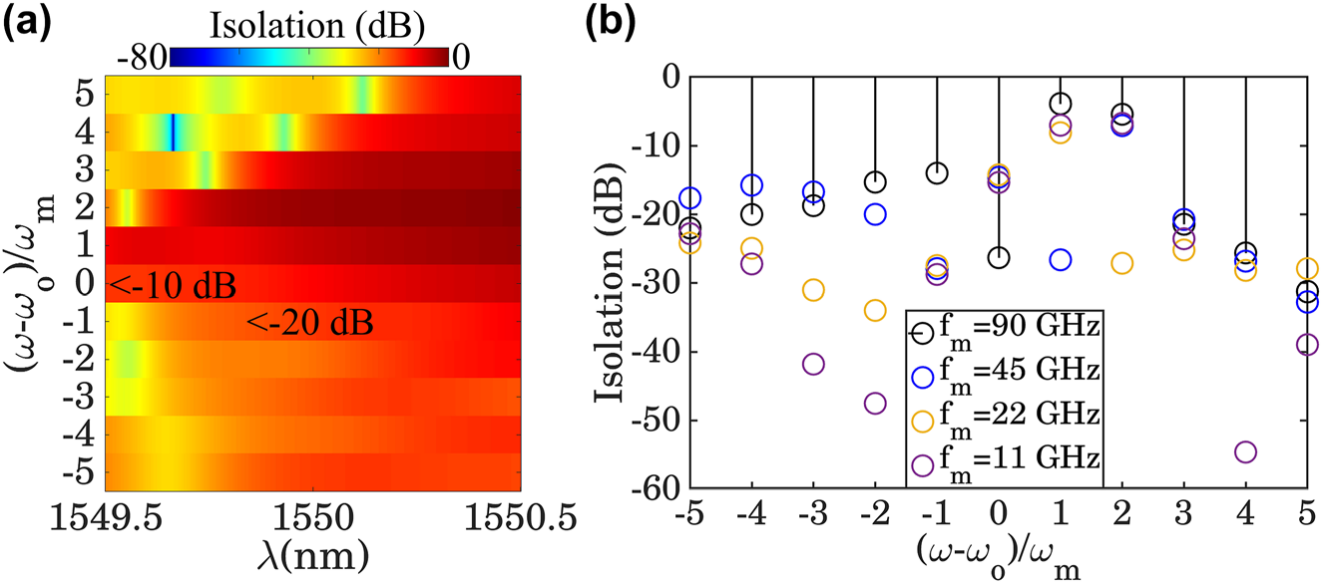
Tunable optical nonreciprocity. (a) The calculated optical power isolation between the existing higher-order harmonics dwelling at port 1 with respect to *ω*_*o*_ + *ω*_m_, as a function of the operating wavelength. On account of the filtering behavior of the static layer, changing the operating wavelength will affect the isolation level from 0 to −80 dB. (b) The dependency of the power isolation between *ω*_*o*_ and *ω*_*o*_ + *ω*_m_ on the modulation frequency, when the operating wavelength is fixed to *λ*_*o*_ = 1549.6 nm.

To investigate the possibility of establishing nonreciprocal responses via the presented purely time-modulated configuration, we set the operating wavelength to *λ*_*o*_ = 1549.6 nm and fix the modulation frequency to *f*_m_ = 20 GHz, which ensures the adiabatic regime of modulation $\left(f_{m}/{\;f}_{o}\approx 10^{-4}\right)$ . The output frequency spectrum of the incident signal in the forward direction that is transmitting from the first port to the second one with the carrier frequency of *ω*_*o*_, is demonstrated in Figure 5a, which clearly exhibits a single line at the fundamental tone with the amplitude of unity. It should be mentioned that Gaussian beam whose width and energy are finite is usually preferred in practice over a plane wave with infinite energy. However, as long as the incoming wave is monochromatic, the optical response of the presented configuration remains unchanged, since the spatial distribution of the incident wave has no effect on the overall response of the structure. Upon the interaction of the light with the static platform, its amplitude drops slightly from unity to almost 0.85; while it’s spectral information remains untouched, as shown in Figure 5b. It should be noted that the decrease in the amplitude of the signal after its interaction with the static layer is merely due to the optical response of the metasurface when the operating wavelength is *λ*_*o*_ = 1549.6 nm. Such a drawback can be compensated by utilizing other ultrahigh-Q metasurfaces that ensure absolute unity transmission in the operating wavelength. As it is depicted in Figure 5e, after interacting with the temporal layer, the frequency component of the incident signal will be distributed to higher-order harmonics, each with different amplitudes. Therefore, since the second port is operating at *ω*_*o*_ + *ω*_m_, a communication link can be established between these two ports in the forward mode. In order to investigate the response of the given configuration in the time-reversal scenario and the amount of isolation level, the signal of Figure 5c, with all of its spectral information, should be sent from the second port to the first one (Figure 5d) and the power of the fundamental tone should be evaluated with respect to the first up-modulated harmonic. Upon interaction of the signal with the time-modulated platform, all of its frequency components undergo another temporal photonic transition, leading to a new output frequency spectrum with different amplitudes, as shown in Figure 5e. As shown in the inset of Figure 2b, the static layer’s filtering behavior is designed such that it can almost totally reflect the signals with the frequencies of *ω*_*o*_ ± *ω*_m_, *ω*_*o*_ ± 2*ω*_m_ and higher. Therefore, once the signal with the output frequency of Figure 5e interacts with the static layer, almost all the power will be reflected in the second port, leading to a negligible amount to receive at the first port, as shown in Figure 5f. According to the calculated results of Figure 5, it is clear that the presented configuration exhibit different optical responses with respect to the direction of illumination, which is a manifestation of a nonreciprocal behavior. In addition, the amount of isolation between the first port (operating at *ω*_*o*_) and the second port (operating at *ω*_*o*_ + *ω*_m_) in the time-reversal scenario via our presented purely temporal configuration is ≈ −15 dB. To the authors’ best knowledge, this is the first time such a high isolation value has been obtained via a purely temporal platform in the NIR regime. However, while in the time-reversal scenario, we have been able to isolate the ports working at *ω*_*o*_ and *ω*_*o*_ + *ω*_m_, the isolation between other higher-order harmonics (shown in Figure 5f) should also be investigated. For this purpose, we have evaluated the optical power isolation between the existing higher-order harmonics at the receiver side (port 1), with respect to *ω*_*o*_ + *ω*_m_ as a function of operating wavelength, and its result is depicted in Figure 6a. As can be seen from this figure, the power isolation level between *ω*_*o*_ + *ω*_m_ and other higher-order harmonics at the receiver depends on the operating wavelength and varies from 0 to −80 dB. The reason why the isolation level changes with respect to the operating wavelength lies at the heart of the filtering behavior of the static layer. In particular, when the wavelength is selected in such a way that after interacting with TMM, the frequency of the higher-order harmonics (i.e., *ω*_*n*_ = *ω*_*o*_ + *nω*_m_) are not falling within the bandwidth of the resonance dip, shown in the inset of Figure 2b, then under time reversal, the static layer becomes transparent to the higher harmonics, leading to lower value of power isolation. However, at the selected operating wavelength of *λ*_*o*_ = 1549.6 nm, the power isolation level between *ω*_*o*_ + *ω*_m_ and other higher-order harmonics changes from ≈ −10 to ≈ −40 dB, which ensures the complete isolation and asymmetrical response of the static layer. It should be mentioned that the underlying concept of this paper dictates isolation levels with higher amounts. However, the existence of undesired higher-order harmonics that are attributed to the limited phase and amplitude modulation affects the level of power isolation. Such a drawback can be ameliorated through the advent of novel materials and/or design paradigms that can lead to higher phase span and lower amplitude modulation. In addition, owing to the tunable features of the time-modulated platform, it is possible to control the isolation level via changing the modulation frequency, as is shown in Figure 6b. In particular, by changing the modulation frequency, the spectral position of the higher-order harmonics changes within the operation bandwidth of the static layer, which subsequently leads specific harmonics to fall inside the resonance dip frequency range. In this perspective, when the modulation frequency changes from 11 to 90 GHz, the power isolation between port 1 and port 2 can reach ≈ −30 dB as demonstrated in Figure 6b. It should be remarked that while changing the modulation frequency can manipulate the power isolation between *ω*_*o*_ and *ω*_*o*_ + *ω*_m_, it will also alter the isolation level between other higher-order harmonics. Such a trade-off should be taken into account when the proposed configuration is ought to be exploited in a realistic experimental setup. Furthermore, it should be noted that while the ideal performance for the presented configuration is obtained based on monochromatic excitation, there might be scenarios wherein the incoming wave has a finite bandwidth around the operating wavelength (i.e.,*λ*_*o*_). In this case, the nonreciprocal response remains unchanged as long as the bandwidth of the incoming beam under time-reversal and after interacting with the temporal layer remains in the close vicinity to the spectral position of the frequency that corresponds to the dip of the static metasurface. Moreover, the presented nonreciprocity of this paper remains unchanged as long as the particular waveform of Figure 4e, which can up-convert the fundamental tone to the first higher order harmonic, is applied to the metasurface. It is also notable to mention that the proposed configuration of this paper can be readily implemented for establishing optical isolation between two ports operating at the same frequency of *ω*_*o*_ [66, 69]. For this purpose, another temporal layer that is subjected to a particular waveform, which can down-modulate the incident tone, should be utilized before the static layer. In this perspective, when the incident wave illuminates from the first port to the configuration, it will firstly be down-modulated to *ω*_−1_ = *ω*_*o*_ − *ω*_m_ which is transparent to the static layer. After passing through the static layer, the transmitted wave interacts with the second temporal layer and undergoes another temporal photonic transition as *ω*_−1_ + *ω*_m_ = *ω*_*o*_. Therefore, the received wave at the second port will have the same frequency as that of the incident wave. Under time-reversal, the incident wave at port 2 has the frequency of *ω*_*o*_, which after interacting with the temporal layer, its frequency will be up-modulated to *ω*_*o*_ + *ω*_m_. On account of the filtering behavior of the static layer, such an incident frequency falls within the resonant dip of the metasurface, which subsequently leads to total reflection of the incident power to port 2 (the received signal will have the frequency of *ω*_+2_ = *ω*_*o*_ + 2*ω*_m_), yielding optical isolation between the two ports. As the final remark, it should be noted that the presented idea of this paper does not rely on any assumptions regarding the shape of the incident signal, and any form of incident wave demonstrates such an asymmetrical behavior with the propounded configuration. Moreover, all the corresponding analysis of this paper was carried out in the weak nonlinear regime of operation, in order to avoid nonlinear effects.

## 5 Conclusions

Since the advent of time-modulated metasurfaces, their frequency mixing property has attracted the attention of the scientific community due to the wealth of applications that they can offer. Based on such a feature of TMMs, we demonstrated the possibility of establishing optical isolation in the NIR regime via a purely temporal configuration. In particular, the presented setup consisted of a time-modulated metasurface and a static high-Q platform that acts as a filter with a sharp optical response. While the refractive indices of the consisting elements of the TMM were optimized in such a way that it can up-convert the incident tone to the first higher-order harmonics, the static layer was designed to be transparent in a broad spectrum except in a narrow band region. It was shown that while the first port is competent in acquiring the transmitted signal in the forward direction, optical power isolation with the level of −15 dB can be established under the time-reversal scenario. We have also calculated the amount of isolation level between other higher-order harmonics and demonstrated that their values change in the range of −10 to −40 dB. The role of modulation frequency on the level of power isolation has also been investigated, and it was shown that the isolation level could reach −30 dB if the modulation frequency increases to 90 GHz. Since this is the first time a nonreciprocal response is obtained in the NIR regime via a pure temporal modulation, we believe the idea of this paper can be of utmost importance in various applications, such as tunable optical isolators.
